# Supplementary material for: Trustworthy AI for medical decisions: Adversarially robust and fair machine learning prediction for Parkinson’s disease
Source: PLoS One. 2026 Feb 23;21(2):e0342062. doi: 10.1371/journal.pone.0342062 (PMC12928579; doi:10.1371/journal.pone.0342062)
Supplement: Attachment — Submitted filename: Response Files.docx [file pone.0342062.s001.docx]

**Response to the Reviewers on “Trustworthy AI for medical decisions: Adversarial robust and fair machine learning prediction for Parkinson’s disease”**

Junaid Muhammad, Mitra Ghergherehchi , Shiraz Ali, Ho Seung Song, Nasir Rahim

Dear editor,

We thank you and the reviewers for your positive comments that helped us a lot to enrich the overall quality of this study. We have considered the reviewer’s comments carefully and tried our best to incorporate all the comments/suggestions to demonstrate the effectiveness of our proposal.

To address each comment carefully, we would like to respond to them point by point. Please note that all updates in the manuscript have been highlighted in red color.

Kind regards,

Authors

**Reviewer’s comment**

The abstract should more clearly state the study’s objectives, dataset characteristics, and the rationale for including adversarial attack analysis.

**Author’s response**

Thank you for your valuable comment.

We have revised the abstract to more clearly state the study’s objectives, describe the PPMI dataset characteristics, and explain the rationale for incorporating adversarial attack analysis. This change enhances the clarity and purpose of our work from the outset.

**Reviewer’s comment**

In Section 3.2.2, several claims—particularly regarding model robustness, federated learning, and explainability—require proper justification and supporting references.

**Author’s response**

Thank you.

In **Section 3.2.2**, we have revised the claim regarding model robustness, federated learning, and explainability by providing proper justification and supporting references from recent literature [98-105]. These additions ensure that the statements are well-supported and grounded in current research, improving the clarity and credibility of the section.

**Reviewer’s comment**

The discussion on bias related to gender, age, and race should be more clearly substantiated with examples and explanations.

**Author’s response**

Thank you.

We have updated the discussion on data bias related to gender, age, and race in **Section 3.2.2** to provide more concrete examples and detailed explanations. This revision ensures that the impact of bias is better substantiated and aligns with the study's objectives.

**Reviewer’s comment**

The mention of "research focused on algorithmic fairness" in Section 3.3 lacks citation, and Section 3.4.2 appears misaligned with its intended focus on group fairness.

**Author’s response**

Thank you.

We have updated **Section 3.3** and included relevant citations supporting the discussion on algorithmic fairness. Additionally, we have revised **Section 3.4.2** to better align with its intended focus on group fairness, ensuring clearer structure and consistency in the manuscript.

**Reviewer’s comment**

A clearer distinction between sections 4.2 and 4.4 is needed as they currently overlap in content.

**Author’s response**

Thank you.

We have merged **Section 4.2** and **Section 4.4**, as they contained redundant content. The revised section now presents the data preprocessing and preparation steps in a more streamlined and cohesive manner, eliminating overlap while maintaining clarity.

**Reviewer’s comment**

Furthermore, the limitations of the experimental setup should be explicitly addressed.

**Author’s response**

We have explicitly addressed the limitations of the experimental setup in **Section 6**. The updated section provides a detailed discussion of the constraints, ensuring a more transparent and comprehensive understanding of the study's limitations.

**Reviewer’s comment**

Key related works and comparisons, particularly those using similar datasets and standard models like SVMs or neural networks, should be included.

**Author’s response**

Thank you.

We have added **Table 7**, which includes a comparison of key related works, particularly those using similar datasets and standard models like SVMs and neural networks. This addition strengthens the contextual foundation of our study and highlights how our approach compares to existing research.

**Reviewer’s comment**

Feature selection choices, especially the exclusion of age and race, should be justified.

**Author’s response**

Thank you.

We have updated **Section 4.1.6** to provide a clearer justification (line 386) for our feature selection choices, specifically regarding sensitive attributes exclusion of age and race. This update explains the rationale behind these decisions, focusing on reducing bias and ensuring fairness in the model's predictions.

**Reviewer’s comment**

Metric inconsistencies should be addressed, with valid ranges and statistical significance testing included.

**Author’s response**

Thank you.

We have addressed the metric inconsistencies in the **Results and Discussion section** (Section 5) by ensuring that all metrics fall within their valid ranges. Additionally, we have included statistical significance testing results to validate the results, providing a more rigorous analysis of model performance.

**Reviewer’s comment**

Imbalances in class distribution should be noted and mitigated appropriately.

**Author’s response**

Thank you.

We have updated **Section 3.2.4** (Data Splitting) to explicitly note the class imbalance in the dataset. Additionally, we have described the methods used to mitigate this imbalance, such as stratified sampling and SMOTE, ensuring a more balanced representation during training and evaluation.

**Reviewer’s comment**

Data availability should align with reproducibility standards, and the ethical treatment of sensitive attributes—such as discretization of age or race—requires clear explanation.

**Author’s response**

Thank you.

We have updated **Section 4.1.1** (Data Collection) to ensure that data availability aligns with reproducibility standards. Additionally, we have provided a clear explanation of the ethical treatment of sensitive attributes, including the rationale behind the discretization of age and race, to maintain transparency and fairness in our approach.

**Reviewer’s comment**

It is important to explicitly note limitations, such as the exclusion of deep learning models, and discuss strategies to manage accuracy-fairness trade-offs in future work.

**Author’s response**

Thank you.

We have noted the limitations of our study, including the exclusion of deep learning models, in **Section 6**. Additionally, we have discussed strategies to manage accuracy-fairness trade-offs in future work, outlining potential directions for incorporating more complex models while maintaining fairness in the predictions.

**Reviewer’s comment**

Language should be polished for grammatical accuracy and reduced complexity.

**Author’s response**

We have carefully proofread the manuscript and revised improve grammatical accuracy and reduce complexity. These changes ensure that the language is clear, concise, and accessible while maintaining the technical rigor of the study.

**Reviewer’s comment**

Formatting and consistency in references, notation, and tables should also be reviewed.

**Author’s response**

Thank you.

We have reviewed and ensured consistency in the formatting, references, notation, and tables throughout the manuscript. All inconsistencies have been addressed, and the formatting now adheres to the journal's guidelines.

**Reviewer’s comment**

The paper would benefit from increased originality and refinement in both content and presentation.

**Author’s response**

Thank you.

We have made significant efforts to increase the originality and refinement of both the content and presentation. The manuscript has been updated to include more detailed justifications, improved clarity, and enhanced structure, ensuring that the contributions are clearly communicated and aligned with the current state of the field.

**Reviewer’s comment**

The authors are encouraged to compare their findings with current state-of-the-art methodologies to better contextualize their contribution.

**Author’s response**

Thank you.

We have included **Table 7**, which compares our findings with current state-of-the-art methodologies. This comparison helps better contextualize our contributions and highlights how our approach aligns with or differs from existing techniques in the field.

**Reviewer’s comment**

The introduction contains an extensive list of contributions, which could be narrowed to emphasize the main innovations.

**Author’s response**

Thank you.

We have revised the **Introduction** section in the manuscript to narrow down the list of contributions, emphasizing the main contributions of the study. This revision enhances the clarity of the manuscript and provides a more focused overview of the key findings.

**Reviewer’s comment**

The concept of fairness is frequently mentioned but not always supported by concrete evidence.

**Author’s response**

Thank you.

We have updated **Section 3** and provided concrete evidence supporting the concept of fairness. Specific examples, metrics, and references to strengthen the discussion and substantiate the fairness-related claims made in the study.

**Reviewer’s comment**

A comparative table summarizing models, datasets, pros, cons, and performance would be a valuable addition.

**Author’s response**

Thank you.

Table 7 has been added to provide a comparative analysis of our findings with those reported in existing literature. The comparison focuses on relevant studies published within the past five years.

**Reviewer’s comment**

Certain sections could be restructured for clarity.

**Author’s response**

We have restructured the relevant sections of the manuscript to improve clarity and flow, especially **section 2,3,4**. The revisions ensure that the content is presented in a more logical and coherent manner, making it easier for readers to follow the study’s key arguments and findings.

**Reviewer’s comment**

Also, the methodology and results sections would benefit from added detail on experimental setup, parameters, and statistical significance.

**Author’s response**

Thank you.

We have expanded **Section 4.5** (Experimental Setup) and **Table 8** and provided more details on the experimental setup and parameters of each model. Additionally, **Section 5** (Results and Discussion) has been updated to include statistical significance testing for key results, enhancing the rigor and interpretability of our findings.

**Reviewer’s comment**

A dedicated discussion section analyzing the results would strengthen the manuscript.

**Author’s response**

We have updated the **Comparative Analysis section** (Section 5.3) in **Results and Discussion** section. This section provides a thorough analysis of the results, comparing our findings with existing methods and highlighting the implications of our study, which strengthens the manuscript’s overall contribution.

**Reviewer’s comment**

Methodological transparency can be improved by detailing fairness mitigation algorithms, feature selection techniques, and adversarial attack parameters.

**Author’s response**

Thank you.

To improve methodological transparency, we have included detailed information about the fairness mitigation algorithms, feature selection techniques, and adversarial attack parameters in **Table 8**. This addition provides a clearer understanding of the methods used and ensures that our approach is reproducible and well-documented.
=====================================================

Reviewer #1:

**Reviewer’s comment**

Your work lacks sufficient originality and does not meet the minimum quality standards of PLOS ONE Journal.

**Author’s response**

Thank you.

We have made significant efforts to increase the originality and refinement of both the content and presentation. The manuscript has been updated to include more detailed justifications, improved clarity, and enhanced structure, ensuring that the contributions are clearly communicated and aligned with the current state of the field.

**Reviewer’s comment**

You need to enhance the English writing in all paper sections. Several statements and paragraphs are not understood.

**Author’s response**

Thank you.

We have carefully reviewed the entire manuscript to improve the clarity, readability, and flow of the language. Also, we have restructured the relevant sections of the manuscript to improve clarity and flow, especially **sections 2,3,4**. The revisions ensure that the content is presented in a more logical and coherent manner, making it easier for readers to follow the study’s key arguments and findings.

**Reviewer’s comment**

Rewrite the abstract section to specify the problem directly and describe your solution in brief.

**Author’s response**

Thank you.

We have revised the abstract section of the proposed study. The updated version of the abstract now clearly states the study’s objectives, describes the PPMI dataset characteristics, and explains the rationale for incorporating adversarial attack analysis. This change enhances the clarity and purpose of our work from the outset.

**Reviewer’s comment**

Compare your results with state-of-the-art approaches.

**Author’s response**

Thank you.

We have updated **Table 7** and compared our results with the state-of-the-art approaches. This comparison provides a clearer context for our findings and highlights the strengths and unique contributions of our methodology relative to existing research.

**Reviewer’s comment**

In Adversarial Robustness section 3.2.2, you draw conclusions without justification. (There is a lot of bias in the information when it comes to gender, age, and race, which shows how these traits are uneven across classes. Compared to gender features, age and race features are more skewed). Explain how bias exists?

**Author’s response**

Thank you.

We have revised **Section 3.2.2** (Adversarial Robustness) and included a detailed explanation of how bias exists in the model and how it affects gender, age, and race features. Specifically:

*Gender bias*: While gender features show imbalance, they are relatively evenly distributed across the dataset. However, adversarial perturbations in gender-based features can still lead to biases in predictions due to disproportionate representation of certain gender groups within the training set.

*Age and Race bias*: The age and race features are more skewed in our dataset, with most PD patients being over 60 years old and a higher proportion of White participants. This uneven distribution of these sensitive attributes introduces bias into the model, as the algorithm may begin to learn spurious correlations between these features and the target class. For example, if the model over-relies on age or race to make predictions, it can lead to discriminatory outcomes when applied to underrepresented groups.

By including such details in the manuscript clarify how adversarial attacks, such as label leakage or poisoning, exacerbate these biases by introducing manipulations that emphasize these skewed features, further reinforcing the unfair treatment of minority groups. This detailed explanation highlights how bias manifests within the data and its impact on fairness metrics, such as Statistical Parity Difference (SPD) and Equal Opportunity Difference (EOD).

**Reviewer’s comment**

In **section 3.2.2** there are conclusions without justification, such as:

Adversarial training is one of the strong training methods that boost model generalization and manipulation resistance.

**Author’s response**

We now include references and explanations that highlight how adversarial training improves both model robustness and generalization by exposing the model to adversarial examples during training. This enables the model to learn not only from clean data but also from perturbed inputs, improving its ability to generalize to real-world, unseen data and making it more resistant to adversarial manipulation. Relevant studies e.g., [102,103,104,10], are cited to substantiate this claim, demonstrating that adversarial training leads to improved performance on both clean and adversarial perturbed test sets.

**Reviewer’s comment**

Resilience added to federated learning systems increases data privacy and model efficiency.

**Author’s response**

We have updated the statement to clarify that adding resilience to federated learning (FL) systems enhances data privacy by keeping data decentralized and improves model efficiency by defending against adversarial attacks. Resilient FL systems can better manage malicious perturbations while maintaining robust performance, ensuring secure and reliable predictions.

**Reviewer’s comment**

Explainable artificial intelligence (XAI) may highlight the evolution and upkeep of robustness.

**Author’s response**

We have updated the statement to clarify that Explainable Artificial Intelligence (XAI) can enhance robustness by providing insights into model decision-making processes. This transparency helps identify vulnerabilities and improve model performance over time, ensuring that the model remains both interpretable and resilient to adversarial attacks.

**Reviewer’s comment**

Furthermore, standardizing model robustness assessment will be a new criterion for healthcare artificial intelligence.

**Author’s response**

Thank you.

We have updated the statement to clarify that standardizing model robustness assessments will become a key criterion for evaluating healthcare AI systems. This approach ensures consistent, reliable performance across different environments and protects against adversarial attacks, fostering trust and safety in healthcare applications.

**Reviewer’s comment**

In section 3.3, you wrote (In research focused on algorithmic fairness, which involves the removal of biases as illustrated in Figure 1) which research do you mean? No reference is given.

**Author’s response**

Thank you.

The reference to research on algorithmic fairness has been updated and appropriately cited [24,25] in a clearer manner **in section 2.4** of the manuscript.

**Reviewer’s comment**

The content of section 3.4.2 Group fairness does not relate to Group fairness; it relates to the model’s assessment.

**Author’s response**

Thank you.

We have updated **Section 3.4.2** to better align with its intended focus. The content has been moved to **Section 3.3.2** (Group Fairness), where it now properly discusses the model’s assessment related to fairness metrics, ensuring clarity and consistency with the rest of the manuscript.

**Reviewer’s comment**

Section 4.2 Data prepossessing and section 4.4 Data preparation have the same contents. What are the differences?

**Author’s response**

Thank you.

We have re-arranged **Section 4.1** to be solely dedicated to Data Preparation, effectively combining the previously redundant **Sections 4.2** (Data Preprocessing) and **Section 4.4** (Data Preparation). The updated content now eliminates overlap, clearly differentiating between data preparation steps and enhancing the clarity of the methodology.

**Reviewer’s comment**

Experiments limitations should be discussed.

**Author’s response**

The limitations of the experiments have been discussed and updated in **Section 6**. This section now addresses key limitations, including the exclusion of deep learning models and the impact of dataset constraints, and outlines strategies for overcoming these challenges in future work.

========================================================

Reviewer #2:

**Reviewer’s comment**

The abstract lacks clarity regarding the objective of using machine learning techniques. Additionally, a description of the dataset is also not given in the abstract. Adversarial attacks are not clear, and why add it to the data?
**Author’s response**

Thank you for your valuable comments.

In response, we have updated the abstract to provide clearer objectives for using machine learning techniques, specifically focusing on their application to Parkinson’s disease prediction. Additionally, we have included a brief description of the PPMI dataset, which is central to our study. We also clarified the role of adversarial attacks, explaining their purpose in testing model robustness and ensuring fairness in the context of healthcare applications. This revision aims to provide a more comprehensive and understandable overview of the study.

**Reviewer’s comment**

In the introduction, the number of primary contributions mentioned is huge; it is better to be reduced to your main contribution only.

**Author’s response**

Thank you.

We have revised the introduction to narrow down the list of primary contributions, focusing on the main innovation of the study. This streamlined presentation highlights the key contribution clearly and concisely, ensuring that the paper remains focused and impactful.

**Reviewer’s comment**

The authors utilize the word fairness many times in many sections without proving if they fairly use it. For example, in the literature review they make, however, there are many articles not included in the study.
**Author’s response**

Thank you.

We have revised the manuscript to substantiate the use of fairness throughout the paper. We have provided concrete examples of how fairness is assessed and integrated into our methodology. Additionally, we have updated the literature review to include key relevant studies that were previously omitted, ensuring a more comprehensive and balanced discussion of fairness in the context of machine learning and healthcare.

**Reviewer’s comment**

in subsection 2.1, "such as those covered here," unclear” pronoun those.”

**Author’s response**

We have updated **subsection 2.1** to clarify the use of the pronoun "those". The statement now explicitly refers to the biases and fairness issues discussed in the section, ensuring greater clarity and eliminating any ambiguity.

**Reviewer’s comment**

In Related Works, it is better to add the most related models that have already been applied to the same dataset of Parkinson. Do any authors utilize other standard ML techniques such as SVM or neural networks?

**Author’s response**

Thank you.

We have added **Table 7** in the **Related Works section**, which now includes comparisons with models that have been applied to the Parkinson’s dataset. We also highlighted the use of standard machine learning techniques, such as SVMs and neural networks, in previous studies, providing a clearer context for how our approach compares to existing methods. Additionally, **Section 5** (Results and Discussion) now includes a detailed analysis of how our findings align with or differ from these techniques.

**Reviewer’s comment**

It is better to add a table with the ML techniques, dataset, pros and cons of each study, and performance.

**Author’s response**

Thank you.

We have updated **Table 7** and included a comprehensive comparison of machine learning techniques, the data set used in each study, their pros and cons, and the performance metrics reported. This updated table helps to clearly contextualize our work by highlighting key differences and similarities with related studies, making it easier for readers to evaluate the relative strengths of various approaches.

**Reviewer’s comment**

Why add this "section 3.3 Mitigate ML bias"? In methodology, just mention the main methods that you are utilizing in your study or that you wish to compare with only.

**Author’s response**

We have updated and merged **Section 3.3** (Mitigate ML Bias) with **Section 3.1** (Fairness and Robustness in Medical AI). This consolidation ensures a more streamlined discussion, providing a comprehensive overview of both fairness and robustness in the context of medical AI, without redundancy. This also enhances the clarity and flow of the manuscript.

**Reviewer’s comment**

3.2.1 Model robustness: In lines 191–192, it is better to add a reference for your claim.

**Author’s response**

Thank you.

We have added the appropriate reference [43,102] in **Section 3.2.1** (Model Robustness), specifically for the claim made in lines 191–192, now in updated version 199-205 line. The updated section now includes relevant citations to support the discussion of model robustness and its importance in adversarial settings.

**Reviewer’s comment**
Add reference for lines 378 and 379: XGBoost and SULOV. Which one did you utilize in your study?

**Author’s response**

We have added the appropriate references for lines 378 and 379 now are 302-405 regarding XGBoost and SULOV. We clarified that our study utilized XGBoost for feature selection, as detailed in citation 70 and 71, and SULOV for redundancy reduction, as referenced in citations 115 and 114. This ensures that the methodologies used are properly cited and aligned with existing research.

**Reviewer’s comment**
In data splitting, there is no reason to make 5-fold cross-validation after the initial separation of the data.

**Author’s response**

We acknowledge the reviewer’s concern regarding the use of 5-fold cross-validation following an 80/20 train-test split. However, our choice was methodologically motivated by the need to achieve reliable class proportions, hyperparameter tuning, variance estimation, and model generalization within the training set, while preserving an untouched hold-out test set for final evaluation. Specifically, the 5-fold cross-validation was applied only on the training portion (80%) to optimize model performance and fairness metrics under various configurations (e.g., different bias mitigation strategies and feature selections). Such a setup is common in fairness-aware ML pipelines to ensure that both accuracy and fairness metrics (e.g., SPD, EOD) are not artifacts of a specific data partition, especially in imbalanced or heterogeneous datasets such as PPMI. This approach is also supported in fairness-sensitive healthcare applications, where reproducibility and generalization are paramount.

**Reviewer’s comment**

In the data collection, the number of features is around 15. Could you please explain the reasoning behind using feature selection?

**Author’s response**

Although the final dataset used in our study includes approximately 15 features, the original dataset contained over 600 features, as detailed in **Supplementary File S1**. Feature selection was therefore a crucial step to filter out noisy, redundant, or weakly predictive variables. Using methods such as SULOV and recursive XGBoost, we identified the most reliable and non-biased features to enhance model performance, reduce overfitting, and ensure fairness across sensitive groups.

**Reviewer’s comment**

And if you used FS, why did you exclude age and race? You can let FS select the most valuable features.

**Author’s response**

Age and race were intentionally excluded from the model's training features to prevent indirect reinforcement of social biases and to align with the principles of fairness-aware machine learning. While feature selection techniques like SULOV and recursive XGBoost were used to identify highly predictive variables, protected attributes such as age and race were deliberately withheld from this process to avoid unfair model behavior. Including them even if selected as predictive—could lead to disparate impact or algorithmic discrimination, which is particularly critical in healthcare applications where ethical considerations and equitable outcomes are paramount.

**Reviewer’s comment**

In the results section,
First, it is better to add in the experiment setup.

**Author’s response**

We have expanded **Section 4.5** (Experimental Setup) and **Table 8** to provide more detailed information on the experimental setup and parameters used. Additionally, **Section 5** (Results and Discussion) has been updated to include statistical significance testing for key results, enhancing the rigor and interpretability of our findings.

**Reviewer’s comment**

The parameters for the decision tree and random forest

**Author’s response**

Thank you.

We have added Table 8, which includes the parameters for both the Decision Tree and Random Forest models. This table provides a clear overview of the hyperparameters used in our study, such as tree depth, minimum samples, and other relevant settings for both models. This addition helps clarify the experimental setup and improves the transparency of the methodology.

**Reviewer’s comment**

Comparing your results with the most recent studies already discussed in your related works is preferable. and it is better to compute the significance of your obtained results.

**Author’s response**

We have updated **Table 7** to include a comparison of our results with the most recent studies discussed in the Related Works section. This table now highlights the differences and similarities between our findings and those of other studies, making it easier to contextualize our contributions within the current state-of-the-art. We have also included statistical significance testing in **Section 5** (Results and Discussion) to validate the obtained results.

**Reviewer’s comment**

In addition, it is better to add a discussion section, which discusses the findings behind the results obtained.

**Author’s response**

Thank you.

The limitations of the experiments have been discussed and updated in **Section 6**. This section addresses key limitations, including the exclusion of deep learning models and the impact of dataset constraints, and outlines strategies for overcoming these challenges in future work.

========================================================

Reviewer #3:

**Reviewer’s comment**

Statistical Analysis and Metric Validity:

**Author’s response**

Address Metric Anomalies: Several tables report implausible values (e.g., FPR >1). Revise calculations and ensure all metrics (FPR, FNR) are within valid ranges (0–1).

**Author’s response**

Thank you.

Upon thorough review, we identified that these anomalies were due to misalignment in the calculation logic and table formatting errors in the reporting phase. All evaluation metrics including FPR, FNR, TPR, and others have now been recalculated and validated to ensure they fall within their theoretical bounds of [0,1], as defined by standard confusion matrix formulations. The updated table (Table 5) now accurately reflect the model’s performance and fair characteristics.

**Reviewer’s comment**

Statistical Significance Testing: To confirm significance, include p-values or confidence intervals for key results (e.g., accuracy drops post-mitigation/adversarial attacks).

**Author’s response**

Thank you.

We have included statistical significance testing in **Section 5** (Results and Discussion). We have now reported p-values and confidence intervals for key results, such as accurate drops after mitigation and adversarial attacks, to confirm the significance of the observed changes and ensure the robustness of our findings.

**Reviewer’s comment**

Class Imbalance: Acknowledge and mitigate the impact of the PD: HC imbalance (≈3:2) using techniques like SMOTE, stratified sampling, or synthetic data generation.

**Author’s response**

We acknowledge the class imbalance in the dataset, where Parkinson’s Disease (PD) cases outnumber healthy controls (HC) at an approximate 3:2 ratio. To mitigate its potential impact on model performance and fairness, we employed stratified 5-fold cross-validation to preserve class distribution during training and validation. Additionally, SMOTE (Synthetic Minority Over-sampling Technique) was applied within the training folds to synthetically balance minority samples without data leakage. This approach has been shown to reduce bias and improve generalization in imbalanced medical datasets. Future work may also explore advanced methods such as adaptive synthetic sampling and class-weighted loss functions for further enhancement.

**Reviewer’s comment**

Methodological Clarity:
Fairness Mitigation Pipeline: Provide explicit details on the "optimized preprocessing fairness mitigation" algorithm (e.g., reweighting, adversarial debiasing). Include hyperparameters and code references if available.

**Author’s response**

Thank you.

We have updated **Table 8** to include explicit details on the optimized preprocessing fairness mitigation algorithm used in our study. We now provide information on techniques such as reweighting and adversarial debiasing, along with the corresponding hyperparameters applied during preprocessing. Additionally, we have included references to the relevant code where applicable to ensure full transparency and reproducibility of the fairness mitigation process.

**Reviewer’s comment**

Feature Selection: Clarify the implementation of SULOV and recursive XGBoost (e.g., library versions, selection criteria).

**Author’s response**

Feature selection was performed using the **Featurewiz library** (*pip install featurewiz == 0.5.0*), which integrates SULOV (Searching for Uncorrelated List of Variables) for multicollinearity reduction and recursive XGBoost for relevance ranking. SULOV filters out redundant features based on correlation thresholds and mutual information, ensuring the remaining features are highly informative and minimally correlated. The recursive XGBoost step then ranks features based on their contribution to classification accuracy, allowing selection of an optimal subset. This two-step method enabled us to retain features that contribute significantly to model performance while improving interpretability and reducing the risk of overfitting.

**Reviewer’s comment**

Adversarial Attacks: Specify perturbation magnitudes (e.g., noise levels in poison attacks) and success rates (e.g., adversarial examples generated)

**Author’s response**

In the poison attack, we modified approximately 5% of the training labels (~43 out of 864 samples) linked to sensitive attributes such as age, gender, and race to simulate a real-world label leakage scenario. Additionally, Gaussian noise with a standard deviation of σ = 0.15 was added to the top three most influential features UPDRS III, MoCA, and GDS-15 to introduce targeted perturbations that disrupt the model’s decision boundaries. For the label leak attack, we synthetically induced a correlation of approximately 0.65 between class labels and sensitive attributes by reassigning 10–15% of labels, creating a biased label distribution favoring majority groups (e.g., age ≥ 60, race = 0). These manipulations resulted in fairness metric degradation in over 80% of test cases, indicating a high adversarial success rate. Both attack types were designed in line with standard protocols for evaluating adversarial robustness in fairness-aware medical AI systems.

**Reviewer’s comment**

Data Availability:

Remove Ambiguity: Delete the redundant "available on request" statement unless legally/ethically justified.

**Author’s response**

We have removed the redundant "available on request" statement regarding data availability, as it was unnecessary. The manuscript now clearly states the data availability policy, ensuring alignment with the journal's guidelines and improving clarity. If there are legal or ethical reasons for restrictions, this will be explicitly stated.

**Reviewer’s comment**

Share Processed Data: Deposit preprocessed/feature-engineered datasets in a public repository (e.g., Zenodo) to ensure reproducibility.

**Author’s response**

We have uploaded preprocessed and feature-engineered datasets in a public repository on GitHub to ensure reproducibility. The selected features used in this study can now be accessed at the following link: Github: <https://github.com/Mjunaid00/Fairness-Aware-Parkinson-s-Disease-.git>

This update enhances the transparency and reproducibility of this work.

**Reviewer’s comment**

Ethical and Bias Considerations:

Sensitive Attribute Binarization: Justify thresholds for discretizing age, race, and gender. For example, clarify how age groups were defined (e.g., <60 vs. ≥60).

**Author’s response**

To facilitate fairness evaluation using parity-based metrics, we binarized sensitive attributes as follows:

- Age was discretized into two groups: <60 and ≥60. This threshold was chosen based on clinical relevance, as age 60 is commonly recognized in neurology as a key cutoff distinguishing early-onset from typical-onset Parkinson’s disease.

[28]. Heinzel, S., Berg, D., Binder, S., Ebersbach, G., Hickstein, L., Herbst, H., ... & Amelung, V. (2018). Do we need to rethink the epidemiology and healthcare utilization of Parkinson's disease in Germany. *Frontiers in neurology*, *9*, 500.

- Race was encoded as 0 for non-White and 1 for White participants. This decision was based on the distribution imbalance within the dataset, where White individuals were the majority group. Binarization allowed us to evaluate racial disparity in a controlled two-group setup.
- Gender was encoded as 0 for female and 1 for male, following the binary format used in the original PPMI dataset. This enabled comparative analysis aligned with standard group fairness metrics such as Statistical Parity Difference (SPD) and Equal Opportunity Difference (EOD).

These binarizations ensured consistency across fairness evaluations while preserving the ethical intent of identifying and addressing disparity across protected demographic groups.

**Reviewer’s comment**

Causal Mechanisms: Discuss how adversarial attacks amplify bias (e.g., label leaks correlating with SPD decline) rather than solely reporting correlations.

**Author’s response**

Adversarial attacks do not merely correlate with fairness degradation; they actively amplify existing biases through targeted disruption of feature-label relationships. For instance, in the label leak attack, sensitive attributes (e.g., race or age) are indirectly encoded into the label space, causing models to learn spurious associations that disproportionately favor majority groups. This manipulation causally inflates disparity metrics such as Statistical Parity Difference (SPD), as models begin to rely on protected attributes for prediction. Similarly, poison attacks introduce misleading patterns in features correlated with protected groups, shifting the decision boundary and increasing false positive or false negative rates for minorities. These interventions reveal causal vulnerabilities in the model, where biased data actively distort fairness outcomes rather than passively reflecting pre-existing imbalances.

**Reviewer’s comment**

Language and Grammar:
Correct grammatical errors (e.g., "data was" → "data were," "prepossessing" → " prepossessing ").

**Author’s response**

Thank you.

We have carefully reviewed the manuscript and corrected all grammatical errors, including changes such as “data was” to “data were” and fixing the typo “prepossessing" to “preprocessing”. These revisions improve the overall clarity and grammatical accuracy of the paper.

**Reviewer’s comment**

Simplify jargon-heavy sections (e.g., "quantum adversarial machine learning") with brief definitions or examples.

**Author’s response**

Thank you.

We have simplified the jargon-heavy sections, including terms like “quantum adversarial machine learning”, by adding brief definitions and examples. For clarity, we now explain that quantum adversarial machine learning (QAML) combines the principles of quantum computing with adversarial defense mechanisms to potentially enhance model robustness. This is illustrated with references to recent works that explore the intersection of quantum computing and adversarial robustness (e.g., cite 36, 37, 38).

**Reviewer’s comment**

Reduce redundancy (e.g., repetitive mentions of "fairness-accuracy tradeoffs" in the Discussion).

**Author’s response**

Thank you.

We have reviewed the Discussion section and reduced redundancy, particularly the repetitive mentions of "fairness-accuracy tradeoffs". The revised section now presents the concept more concisely, improving the flow and readability while maintaining the depth of the analysis.

**Reviewer’s comment**

Formatting Consistency:

Ensure uniform citation style (e.g., "[13]" vs "as shown in [13]").

**Author’s response**

Thank you.

We have updated the citations to ensure uniformity in style throughout the manuscript. All citations are now consistently formatted, either as "[13]" or "as shown in [13]", following the required citation style for the journal. This revision enhances the manuscript's clarity and adherence to formatting guidelines.

**Reviewer’s comment**

Standardize mathematical notation (e.g., italics for variables: FPR vs. FPR).

**Author’s response**

Thank you.

We have standardized the mathematical notation throughout the manuscript, ensuring that all variables (e.g., FPR, TPR) are written in italics for consistency and clarity. This revision improves the overall presentation and follows standard conventions for mathematical expressions in academic writing.

**Reviewer’s comment**

Result Presentation:
o Tables 3–6: Reformat to avoid split tables across pages and ensure alignment

**Author’s response**

Thank you. We have reformatted **Tables 3–6** to avoid splitting across pages and ensured proper alignment for better readability. These updates ensure that the tables are fully contained within a single page, improving the overall presentation and flow of the manuscript.

**Reviewer’s comment**

Figures: Improve resolution and label clarity (e.g., axis labels in Figures 5–8).

**Author’s response**

Thank you.

We have improved the resolution and label clarity for Figures 5–8, specifically enhancing the axis labels and ensuring that all visual elements are sharp and legible. These updates contribute to a clearer and more professional presentation of the figures in the manuscript.

**Reviewer’s comment**

Discussion Limitations:

o Explicitly state the exclusion of deep learning models as a limitation and its impact on generalizability.

o Discuss potential solutions to accuracy-fairness trade-offs (e.g., hybrid models, post-hoc calibration

**Author’s response**

Thank you.

For both points, we have updated **Section 6** (Limitations and Future Work) to provide a more thorough discussion of the limitations of the study. This section now includes a detailed analysis of the experimental constraints, such as the exclusion of deep learning models and other challenges, along with strategies for addressing these limitations in future work.
