# Supplementary material for: Trustworthy AI for medical decisions: Adversarially robust and fair machine learning prediction for Parkinson’s disease
Source: PLoS One. 2026 Feb 23;21(2):e0342062. doi: 10.1371/journal.pone.0342062 (PMC12928579; doi:10.1371/journal.pone.0342062)
Supplement: Attachment — Submitted filename: Response to Reviewers.docx [file pone.0342062.s002.docx]

**Response to the Reviewers on “Trustworthy AI for medical decisions: Adversarial robust and fair machine learning prediction for Parkinson’s disease”**

Junaid Muhammad, Mitra Ghergherehchi, Shiraz Ali, Ho Seung Song, Nasir Rahim

Dear editor,

We sincerely thank you and the reviewers for your positive comments that helped us to improve the quality of this study. We carefully considered all the feedback and have incorporated the reviewer’s suggestions to strengthen the manuscript and better demonstrate the effectiveness of our proposal.

To address each comment carefully, we provide a detailed point-by-point response to each comment. Please note that all updates in the manuscript have been highlighted in red.

Best regards,

Authors

**Reviewer #2**

**Reviewer’s comment** 
1. Abstract: The methodology remains unclear. The authors are encouraged to clarify the approach used in the study.

**Author’s Response:**

We sincerely thank the reviewer for their valuable comment.

To improve clarity, we revised the Abstract to explicitly outline the methodological steps followed in our study. Specifically, we now emphasize that we (i) created a baseline with raw data, (ii) applied feature engineering and data balancing methods to mitigate preprocessing bias, (iii) trained fairness-optimized Decision Tree (DT) and Random Forest (RF) classifiers, and (iv) evaluated adversarial robustness using poison and label-leak attacks. Performance was assessed using standard metrics (accuracy, precision, recall, F1-score) and fairness was evaluated using Statistical Parity Difference (SPD) and Equal Opportunity Difference (EOD).

**Reviewer’s comment :**
2. Introduction: The introduction includes a list of six primary contributions, which is quite extensive. It is recommended that the authors narrow it down to the single most significant and novel contribution of the study.

**Author’s response:**
Thank you.

We have revised the list of six contributions into one consolidated statement to highlight the most novel aspect of our work.

**Reviewer’s comment**

3. Table 7: This table should be relocated to the literature review section. Additionally, the authors should analyze and discuss the reviewed papers listed in Table 7 within the context of the literature review.

**Author’s response:**
Thank you.

In the revised version of the manuscript, we have relocated Table 7 from the **Results** section to the **Related Work section (Section 2)**. We also added a dedicated discussion paragraph describing the table. Specifically, we highlight that while prior studies (2021–2025) have achieved high diagnostic accuracy with CNN, SVM, Random Forest, and hybrid models, they largely overlook fairness mitigation and adversarial robustness. Our study addresses this gap by integrating fairness-aware preprocessing with robustness testing under adversarial conditions. This repositioning strengthens the literature review and clarifies the novelty of our contribution.

**Reviewer’s comment:**

4. Results Section: It would be beneficial to add a discussion section to interpret and contextualize the findings.

**Author’s response:**
Thank you.

In the revised manuscript, we have added a dedicated **6. Discussion section** immediately after the Results and before the Limitations. This section interprets the findings, contextualizes them in relation to existing studies, and emphasizes their implications for medical AI. We specifically highlight the trade-off between fairness and accuracy, the varying effectiveness of bias mitigation across models, and the heightened vulnerabilities revealed under adversarial attacks. We also situate our work within the broader literature on fairness and trustworthy AI.

The study's findings suggest that, albeit at a lower raw accuracy, fairness-aware preprocessing approaches can dramatically reduce demographic bias in Parkinson's disease prediction. These revelations will have far-reaching implications for clinical practice. We provide a roadmap for developing more dependable AI systems in healthcare by demonstrating that adversarial evaluation can reveal hidden flaws and fairness-aware preprocessing can reduce biases in Parkinson's disease diagnosis.

5. The authors are also advised to perform statistical tests to assess the significance of the results, especially in comparison with the most recent studies listed in Table 7.

Response:
Thank you.

In the revised version of the manuscript, we have included a dedicated section **5.3 Evaluation of Fairness Statistical Tests** which presents a paired t-tests analysis to statistically assess the significance of our findings. These tests were applied to compare model performance before and after fairness preprocessing, as well as under adversarial attacks.

**Reviewer #3:**

We thank the reviewer for accepting our work.
