# Supplementary material for: Trustworthy AI for medical decisions: Adversarially robust and fair machine learning prediction for Parkinson’s disease
Source: PLoS One. 2026 Feb 23;21(2):e0342062. doi: 10.1371/journal.pone.0342062 (PMC12928579; doi:10.1371/journal.pone.0342062)
Supplement: Attachment — Submitted filename: Response_to_Reviewers_auresp_3.docx [file pone.0342062.s003.docx]

**Response to the Reviewers on “Trustworthy AI for medical decisions: Adversarial robust and fair machine learning prediction for Parkinson’s disease”**

Junaid Muhammad, Mitra Ghergherehchi, Shiraz Ali, Ho Seung Song, Nasir Rahim

Dear editor,

We sincerely thank you and the reviewers for your positive comments that helped us to improve the quality of this study. We carefully considered all the feedback and have incorporated the reviewer’s suggestions to strengthen the manuscript and better demonstrate the effectiveness of our proposal.

To address each comment carefully, we provide a detailed point-by-point response to each comment. Please note that all updates in the manuscript have been highlighted in red.

Best regards,

Authors

**Reviewe’s Comments:** Direct, like-for-like baselines on your splits. The SoTA table is useful, but adding head-to-head results (accuracy and fairness metrics) for at least a strong classical model (e.g., XGBoost/SVM) and one representative DL baseline to match the works you cite.

**Author’s response:**

Thank you for your valuable comments

We agree with the need for head-to-head baselines on our exact splits. Accordingly, we trained and evaluated a list of classifiers including Decision Tree Classifier, Random Forest, LGBM, Extra Trees Classifier, Logistic Regression, KNeighbors, SVC, AdaBoost, Gaussian NB, and SGD Classifier under the same preprocessing, features, and folds. We now report like-for-like results for a strong classical model (i.e., Random Forest) and a strong tree ensemble (Decision Tree) with Accuracy, F1, AUROC, SPD, and EOD as mean ± 95% CI over 10-fold CV (global seed 4765416; fold assignments and code in the repository). Final chosen hyperparameters (not ranges) are listed in Table 9.

However, a representative DL baseline would require DL-specific mitigation, training regimes, and tuning that required detail experiments and exploration; we have clarified this in Methods/Limitations section and will extend our study with a DL model and DL-appropriate fairness methods on the same splits and metrics in a follow-up. But we have added a representative DL baseline to compare the published in this study.

**Reviewe’s Comments:** Fairness method baselines. Compare “optimized preprocessing” against at least one in-processing (e.g., adversarial debiasing) or post-processing (e.g., equalized odds) approach on the same pipeline.

**Author’s response :**

Our study intentionally focuses on the **impact of optimized preprocessing** on tabular ML under label poisoning/leakage, holding the learning algorithm fixed to isolate effects. Adding **in-processing** (e.g., adversarial debiasing or reductions with constraints) and **post-processing** (e.g., equalized-odds thresholding) would introduce model-dependent constraints and substantial hyperparameter/search complexity that is beyond a minor revision and would confound attribution to preprocessing. Also, we have clarified this scope in the **Methods** and **Limitations section**.

**Reviewe’s Comments:** Reproducibility details. Report the final chosen hyperparameters (not just ranges), random seeds, and fold assignments; and provide code or a repository link/script to reproduce preprocessing, fairness metrics, and attack generation (poison rate, leakage construction).

**Author’s response:**

We have updated the paper for reproducibility and robustness. In the Data Availability section, we now provide the public link of our GitHub repository containing the full pipeline of our proposed experimentation workflow. All results are deterministic using a fixed global seed 4765416 with 10-fold cross-validation and provided fold assignments. Table 9 has been revised to report on the final chosen hyperparameters for Decision Tree and Random Forest.

**Reviewe’s Comments:** Generalization check (lightweight). If external validation isn’t feasible now, add a sensitivity analysis (e.g., varying poison rate beyond 5% and reporting CIs across folds) to strengthen the robustness story you’re telling.

**Author’s response:**

To strengthen the robustness claims in our proposed approach, we added a **poison-rate sensitivity analysis** on our exact splits and pipeline, varying (p \in {0,5,10,15,20}%). For each (p), we report **mean ± 95% CI** across the 10 folds for **Accuracy, F1, AUROC**, and fairness gaps (**SPD**, **EOD**). Poisoning is applied **train-only**, via stratified label flips (uniform within class × sensitive-group strata) under the fixed seed. As (p) increases, we observe a **monotonic decline** in utility and **widening** fairness gaps, with **Random Forest** consistently degrading **less** than a single **Decision Tree** quantifying robustness directly in our setting and addressing the editor’s generalization request without external validation data.
