# Supplementary material for: Trustworthy AI for medical decisions: Adversarially robust and fair machine learning prediction for Parkinson’s disease
Source: PLoS One. 2026 Feb 23;21(2):e0342062. doi: 10.1371/journal.pone.0342062 (PMC12928579; doi:10.1371/journal.pone.0342062)
Supplement: Attachment — Submitted filename: Response_to_Reviewers_auresp_4.docx [file pone.0342062.s004.docx]

**Response to the Reviewers on “Trustworthy AI for medical decisions: Adversarial robust and fair machine learning prediction for Parkinson’s disease”**

Junaid Muhammad, Mitra Ghergherehchi, Shiraz Ali, Ho Seung Song, Nasir Rahim

Dear editor,

We sincerely thank you and the reviewers for your positive comments that helped us to improve the quality of this study. We carefully considered reviewer’s comments to strengthen the manuscript and better demonstrate the effectiveness of our proposal.

To address each comment carefully, we provide a detailed point-by-point response to each comment. Please note that all updates in the manuscript have been highlighted in red.

Best regards,

Authors

**Reviewer #2:** All comments have been addressed.

**Author’s response:**

Thank you for accepting our work

**Reviewer #4:**

**Reviewer’s Comment:**

Some sentences in the abstract are long and dense. Consider breaking them into shorter statements to enhance readability.

**Author’s response:**

Thank you for your valuable comments.

We have revised the Abstract where long and dense sentences have been broken into shorter, clearer, and meaningful statements to enhance readability**.**

**Reviewer’s Comment:**

The introduction contains abrupt transitions. Adding linking sentences would improve narrative flow.

**Author’s response:**

Thank you.

We have updated the flow in introduction and better readability, connectivity in overall section.

Section 1 “Introduction” Lines 12–15 have been restructured for improved clarity and flow.

**Reviewer’s Comment:**

The early introduction lists PD symptoms with several citations grouped together. Consider integrating citations more smoothly after each relevant clinical statement.

**Author’s response:**

Thank you.

PD symptoms are now presented with citations integrated immediately after each relevant clinical statement, ensuring smoother and more precise scholarly attribution.

We made updates in “**Introduction**”, “**Related work**” and “**Limitations and future work**” in the manuscript.

**Reviewer’s Comments:**

The description of the PPMI dataset's demographics could be expanded slightly for clarity.

**Author’s response:**

Thank you.

We have revised and expanded description of the PPMI dataset’s demographics, written to improve clarity and transparency while aligning with the content already present in your manuscript (e.g., Table 3 and Section 4.1.1). This version adds contextual detail about participant composition and justifies demographic categorization choices.

**Reviewer’s Comment:**

SPD and EOD are introduced later in the abstract without explanation. Either define them briefly or ensure they appear earlier in the text before being referenced.

**Author’s response:**

Thank you.

In the revised abstract, SPD (Statistical Parity Difference) and EOD (Equal Opportunity Difference) are now introduced with brief, clear explanations at their first mention to ensure accessibility and clarity for all readers.

**Reviewer’s Comment:**

The methodology appears scattered across subsections. Adding a short roadmap paragraph at the beginning of the Methods section would help guide the reader.

**Author’s response:**

A roadmap paragraph has been added at the beginning of the **Materials and Methods** section to clearly outline the structure and guide the reader through the subsections.

**Reviewer’s Comment:**

Ensure every figure and table is explicitly cited in the text in sequential order.

**Author’s response:**

Thank you.

We have reviewed the in-text citations of all figures and tables and identified discrepancies relative to sequential order and explicit mention.

Update: Table 1 has been properly cited in the text and verified for accuracy and consistency.

**Reviewer’s Comment:**

There are occasional grammatical issues.

**Author’s response:**

Thank you.

We thoroughly revised our manuscript and corrected all grammatical mistakes.

**Reviewer’s Comment:**

The conclusion summarizes findings but could benefit from a clearer statement of the study’s practical impact for clinicians and system designers.

**Author’s response:**

Thank you.

We have added a clearer statement to the Conclusion section highlighting the practical impact of our findings for clinicians and AI system designers

**Reviewer’s Comment:**

Although the results are strong, the manuscript would benefit from a brief section acknowledging limitations and future research directions.

**Author’s response:**

Thank you.

We have added a dedicated section “**Section 7”** to the manuscript which acknowledge the study’s limitations and outlining directions for future research.
